# Paclobutrazol induces tolerance in tomato to deficit irrigation through diversified effects on plant morphology, physiology and metabolism

**DOI:** 10.1038/srep39321

**Published:** 2016-12-22

**Authors:** Sikander Pal, Jiangsan Zhao, Asif Khan, Narendra Singh Yadav, Albert Batushansky, Simon Barak, Boris Rewald, Aaron Fait, Naftali Lazarovitch, Shimon Rachmilevitch

**Affiliations:** 1 Jacob Blaustein Institutes for Desert Research, Sede Boqer Campus, Ben-Gurion University of the Negev, Israel

## Abstract

Dwindling water resources combined with meeting the demands for food security require maximizing water use efficiency (WUE) both in rainfed and irrigated agriculture. In this regard, deficit irrigation (DI), defined as the administration of water below full crop-water requirements (evapotranspiration), is a valuable practice to contain irrigation water use. In this study, the mechanism of paclobutrazol (Pbz)-mediated improvement in tolerance to water deficit in tomato was thoroughly investigated. Tomato plants were subjected to normal irrigated and deficit irrigated conditions plus Pbz application (0.8 and 1.6 ppm). A comprehensive morpho-physiological, metabolomics and molecular analysis was undertaken. Findings revealed that Pbz application reduced plant height, improved stem diameter and leaf number, altered root architecture, enhanced photosynthetic rates and WUE of tomato plants under deficit irrigation. Pbz differentially induced expression of genes and accumulation of metabolites of the tricarboxylic acid (TCA) cycle, γ-aminobutyric acid (GABA-shunt pathway), glutathione ascorbate (GSH-ASC)-cycle, cell wall and sugar metabolism, abscisic acid (ABA), spermidine (Spd) content and expression of an aquaporin (AP) protein under deficit irrigation. Our results suggest that Pbz application could significantly improve tolerance in tomato plants under limited water availability through selective changes in morpho-physiology and induction of stress-related molecular processes.

Among horticultural crops, tomato (*Solanum lycopersicum* L.) is one of the most important cash crops cultivated throughout the world^1^. The crop is grown over an area of 5 × 10^6^ ha of arable land worldwide, with an annual production equaling 153 × 10^6^ t of fresh tomato^2^. Tomato yields are dependent upon several genetic, physiological and environmental factors, amongst which drought stress is known to severely hinder tomato productivity^1^. Deficit irrigation is an irrigation regime whereby water supply is lowered below maximum levels and mild stress is permitted with nominal effects on yield. Such a practice is cost-effective, allowing optimal use of allocated water and for production of cash crops helping farmers optimize economic gains^3^. However, this practice requires clear knowledge of crop response to water as drought tolerance differs substantially with species, cultivar and stage of growth^4^,^5^. Almost all commercial tomato cultivars are drought sensitive, either in their developmental stages or during seed germination or seedling establishment. Drought impedes plant growth *via* direct effects on cell division and expansion^6^, and perturbs ion balance and induces senescence^6^. Furthermore, drought leads to the production of reactive oxygen species (ROS), which are highly destructive to lipids, nucleic acids and proteins^7^. Plants respond to a drought episode in several ways, such as stomatal closure, reduced rates of net and gross carbon dioxide (CO_2_) uptake and release from photorespiration, reduced transpiration rates, and massive changes in gene expression leading to the stimulation of the antioxidant system and metabolomic reflux^4^,^7^. Membrane damage, reduced hydraulic conductivity of the leaf vascular system, and a decrease in photosystem II (PSII) electron transport, but enhanced non-photochemical quenching (NPQ)^7^, have been observed under water deficit conditions. It is widely accepted that endogenous plant tolerance mechanisms are generally incapable of completely preventing the deleterious effects of water deficit conditions thus exhibiting stunted growth, poor nutritional quality and reduced yield^7^. During water deficit, stomatal closure is linked with enhanced levels of ABA, which in turn reduces the activity of aquaporins (water channel proteins)^8^. In order to cope with increasing water demand, depth of the root system and stomatal control of water use has been shown to improve drought tolerance in *Coffea canephora*^9^.

Water deficit or drought stress is diligently regulated *via* active participation of ABA, jasmonic acid, salicylic acid, auxins (Aux) and brassinosteroids^10^,^11^,^12^,^13^,^14^,^15^.

Extensive metabolite profiling of crop plants under drought or low water availability has indicated a major shift in the metabolome, a change likely to be associated with improvement in drought tolerance^16^,^17^. As such, metabolomic events under water deficit are guided *via* differential expression of numerous genes involved in the regulation of plant metabolism^18^,^19^. Hence, a strategy that affects the transcriptome and metabolome to induce drought or water deficit tolerance mechanisms could provide a successful approach to enhance plant response to water stress.

Genetic engineering has helped in improving the drought tolerance of tomato cultivars^20^,^21^ although negative public opinion has triggered a debate^20^ preventing its further use. In lieu of genetic engineering, exogenous application of phytohormones has emerged as an alternative approach for strengthening and improving plant tolerance to drought, without altering its genetic makeup^22^,^23^. In recent years, use of pesticides and fungicides, such as Paclobutrazol (Pbz), has shown a potential for improving crop drought tolerance^24^. In general, Pbz is used extensively to control insect and fungal attacks on crops *via* targeting the ecdysis of insects and fungal sterols^25^. The anti-gibberellin (GA) actions of Pbz have been well documented in plants; for example, plants treated with Pbz exhibit stunted growth due to reduced GA endogenous levels^25^. Previous investigations have indicated a role of Pbz in improving the drought tolerance of crop plants, but until now, its mode of action has remained largely unknown. Pbz application has shown positive impact on drought tolerance potential of crop plants, but lack of in-depth understanding about Pbz mode of operation under drought stress limits their application in crop plants. The present study was designed to determine the effects of Pbz on the tolerance of tomato plants under a water deficit regime. To achieve the said objective, a comprehensive investigation was undertaken to elucidate the mechanisms of Pbz-mediated water deficit tolerance using whole plant (physiological) and molecular approaches.

## Results and Discussion

### Pbz induces morphological adaptations that enhance tolerance to deficit irrigation

**Figure 1:**
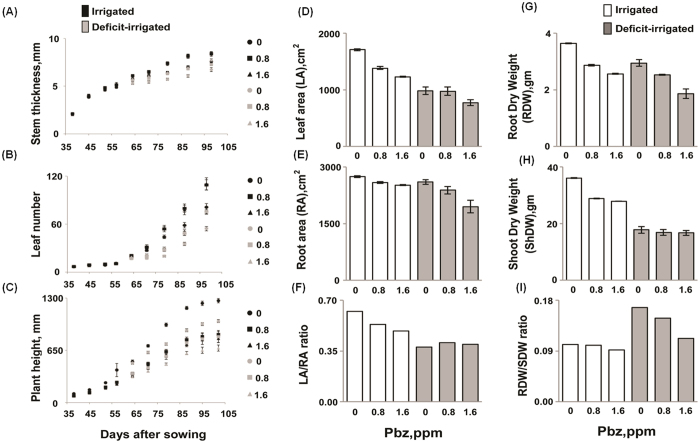
Pbz induced morphological adaptations under deficit irrigation. Effects of PBZ application (0, 0.8 and 1.6 ppm) in irrigated and deficit-irrigated tomato plants grown over a period of 105-days on the morphological parameters including (**A**) stem thickness, (**B**) leaf number, (**C**) plant height, (**D**) leaf area, (**E**) root area, (**F**) leaf area(LA)/root area (RA) ratio, (**G**) root dry weight (RDW), (**H**) shoot dry weight (ShDW) and (I) RDW/ShDW ratio.

Pbz application resulted in a reduction in tomato plant height irrespective of growth conditions (Fig. 1C) in line with previous published reports^26^. Reduced size in Pbz-treated plants may be linked with its inhibitory action on the GAs, which are involved in cell division. Pbz application also increased stem width and leaf number in all the conditions tested (Fig. 1A,B). These observations are in accordance with previous work in tomato and other crop plants^27^. Pbz (at 1.6 ppm) significantly reduced leaf area (LA) (28% and 24%) in both irrigated and deficit irrigated conditions (Fig. 1D). Nevertheless the effect of Pbz on tomato roots was a reduction in root area (RA) (at 1.6 ppm under deficit irrigation) (Fig. 1E). Integrating leaf and root observations, Pbz application led to decreased LA/RA ratio in irrigated plants (Fig. 1F). The Pbz-induced change in LA under deficit irrigation may be linked to improved water use efficiency (WUE) (Table 1) 28. Water use efficiency (WUE) increased in 0.8 I and 1.6 I plants as compared to irrigated control. However in case of deficit irrigation, the WUE increased only in 0.8 DI plants. The 0.8 DI plants have WUE close to irrigated control plants, which suggest that the Pbz induced higher WUE in deficit irrigated plants affording them improved WUE as irrigated control plants (Table 1).

### Biomass

Applications of Pbz (0.8 and 1.6 ppm) reduced tomato shoot dry weight (ShDW) and root dry weight (RDW) considerably (Fig. 1G,H). No significant change in RDW/ShDW ratio was observed in irrigated plants (Fig. 1I). In contrast, under deficit irrigation, Pbz (at 1.6 ppm) the RDW/ShDW ratio decreased significantly (67%) compared to the control (Fig. 1I), not observed in previous reports^27^,^29^. Changes in the RDW/ShDW ratio have been linked to the Pbz-induced inhibition of GA biosynthesis leading to reduced shoot growth^30^. Pbz application under irrigated conditions lowered relative growth rate (RGR) by 0.7-fold (0.8 ppm). Conversely, this effect of Pbz was overturned during deficit irrigation revealing an increase in RGR by 0.6-fold (1.6 ppm) compared with control plants (Fig. S1).

### Pbz application and physiological parameters

In the current study, chlorophyll content increased in both irrigated and deficit irrigated Pbz treated plants as compared to their respective control (without Pbz). In Pbz treated irrigated plants, the maximum chlorophyll content was observed at 77d, thereafter it gradually declined, while in deficit irrigated Pbz treated plants the maximum chlorophyll content (slightly higher than irrigated plants) was observed at 92d, declining thereafter (Fig. S2). This data demonstrates that Pbz enhanced the chlorophyll content independent of water stress stress in both irrigated and deficit irrigated plants. At 77d, DI+Pbz plants had chlorophyll content equal to irrigated plants, which suggest that Pbz compensates deficit irrigation induced reduction in chlorophyll content by maintaining higher chlorophyll content in DI plants and thus affords them better photosynthesis similar to irrigated plants (Fig. S2). Consequently, Pbz application increased photosynthesis in tomato leaves (Table 1). These observations are in agreement with previous findings^31^,^32^. The mechanism of NPQ is used by plants to guard themselves against adverse effects of stress. NPQ is decreased in Pbz treated irrigated plants (I+Pbz) relative to irrigated control plants (I) while slightly increased in Pbz treated deficit irrigated plants (DI+Pbz) as compared to deficit irrigated (DI) control plants (Table 1). DI+ Pbz plants maintain NPQ equal (slightly increased) to irrigated control plants (Table 1), thus application of Pbz promotes the maintenance of higher NPQ similar to irrigated plants and may helps to protect them from the adverse effects of deficit irrigation stress. Pbz-mediated enhancement of NPQ could have enabled the plants to neutralize excess incident energy through heat dissipation^33^. While higher rates of photosynthesis in the Pbz-treated plants (Table 1) could be linked with leaf structural adaptations, such as higher Chl index and reduced specific leaf area (SLA) (Figs S2 and S3). For example, thicker leaves in combination with high Chl content lead to higher photosynthetic rate in *Catharanthus roseus*^34^. Stomatal conductance increased in DI+Pbz plants as compared to DI control plants. In case of irrigation, Pbz supplemented plants also showed slightly increased stomatal conductance relative to control irrigated plants (Table 1). Application of Pbz increased electron transport rate (ETR) irrespective of the growth conditions (Table 1). The higher stomatal conductance and ETR in the Pbz-treated plants could be attributed to increased cytokinins^35^.

### Pbz affects ABA metabolism and aquaporin expression in tomato

**Figure 2:**
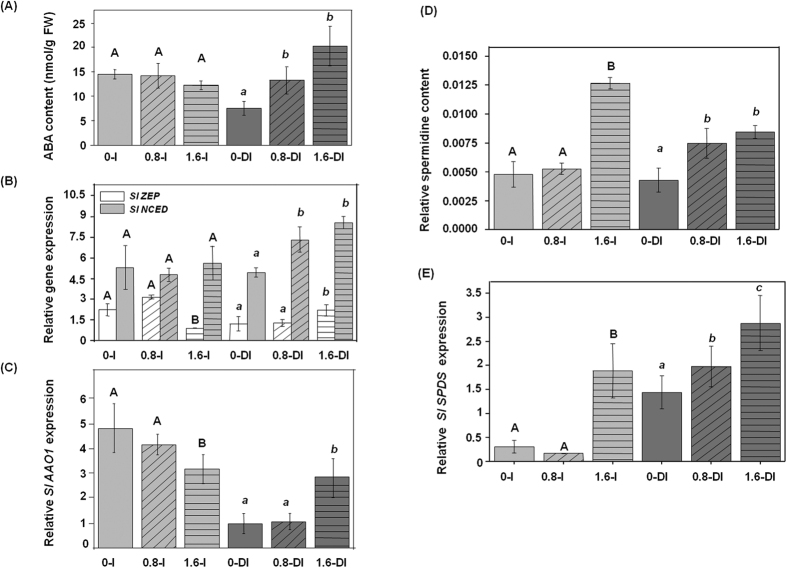
Pbz application modulates endogenous abscisic acid and spermidine contents. Effects of PBZ application (0, 0.8 and 1.6 ppm) in irrigated (I) and deficit-irrigated (DI) tomato plants on the endogenous content of (**A**) abscisic acid (ABA) and expression of ABA biosynthesis genes (**B**,**C**) *SlZEP, SlNCED and SlAAO1*, and relative content of (**D**) spermidine (Spd) and expression of (**E**) spermidine synthase gene (*SlSPDS*) in the leaf tissue of 105-days old tomato plants. Letters (**A**,**B** and a,b,c) indicate significant differences (one way ANOVA, p < 0.05) from the control in irrigated and deficit-irrigated conditions, respectively.

ABA is a key player in a plant’s drought responses, synthesized rapidly at the onset of stress; it affects stomatal closure and root to shoot (R/S) ratio, and consequently the plant’s water status. Endogenous ABA level has been linked with the activity of aquaporins, which play crucial roles in water uptake in plants^36^. To study the influence of Pbz application (0.8 and 1.6 ppm) on ABA metabolism we studied ABA content and expression of ABA biosynthesis genes under irrigated (I) and deficit-irrigated (DI) conditions. The DI+Pbz treated plants showed significantly higher accumulation of ABA content as compared to DI control plants. The irrigated plants did not show enhanced ABA synthesis in the presence of Pbz (Fig. 2A). Further, this increased ABA content in DI+Pbz plants is supported by up-regulation of ABA biosynthesis genes (*SlZEP, SlNCED* and *SlAAO1*) in DI+Pbz plants as compared to DI control plants (Fig. 2B,C). Similarly, the unchanged ABA content in I and I+Pbz plants is supported by expression analysis of ABA biosynthesis genes (Fig. 2B,C). This data supports that Pbz mediated change in ABA content is dependent on irrigation. The one possible reason could be already enhanced GABA signaling in DI plants which leads to activation of Ca^2+^ signaling pathways and ultimately the enhanced activity of ABA biosynthesis pathway. In contrast to previous studies, the control DI plants showed slightly low ABA content as compared to irrigated control plants (Fig. 2A). The main cause behind this unchanged (slightly low) ABA content in deficit irrigated (DI) as compared to irrigated plants may lie in our experimental procedure as well as advanced developmental stage of plants. There are several reports depicting short exposure (from few days to two weeks) of complete drought stress (no watering) inducing the ABA content rapidly. However, there are no reports depicting longer effect (more than one month) of drought stress in older plants in existing literature. In our study, we performed deficit irrigation (70% of the evaporative demand) instead of complete withdrawal of irrigation. Another considerable difference from previous studies is that we started deficit irrigation in older plants (50-days-old) and continued it for up to 105-days. Further, this reduced ABA content in deficit irrigated plants is supported by down regulation of ABA biosynthesis genes (*SlZEP, SlNCED* and *SlAAO1*) in deficit irrigated plants as compared to irrigated plants (Fig. 2B,C). The 0.8 and 1.6 DI plants showed significantly higher ABA contents compared to DI control plants (Fig. 2A). Further, physiological analysis revealed no significant change in shoot dry weight in 0.8 and 1.6 DI plants relative to DI control plants (Fig. 1H). However, leaf area reduced significantly in 1.6 DI plants as compared to DI control plants (Fig. 1D). Surprisingly, the root dry weight significantly reduced in 0.8 and 1.6 DI plants than DI control plants (Fig. 1G). The deficit irrigated control plants (0 DI) exhibited higher RDW/ShDW ratio compared to 0.8 DI and 1.6 DI plants (Fig. 1I). The 1.6 DI plants showed lower RDW/ShDW ratio which is very close to irrigated plants (Fig. 1G). In conclusion, the increased ABA content in 1.6 DI plants helps to sustain better physiological status similar to that of irrigated plants.

Water deficit is often supplemented by a secondary osmotic/oxidative stress in plants. At this point the concentration of ABA builds up facilitating re-dehydration and water uptake in plants *via* aquaporins^37^,^38^. We therefore analyzed the expression of tomato aquaporin gene *TIP2*, (*Sl tonoplast intrinsic protein2*) as well as protein PIP2-7 (Plasma membrane intrinsic protein 2–7), under irrigated and water deficit conditions. Pbz at both 0.8 and 1.6 ppm concentration significantly affected aquaporin (gene and protein) expression compared to control plants supporting a coordinated up-regulation of ABA and aquaporin levels under water deficit (Fig. S4). Western blot analysis showed an elevated PIP2-7 in 0.8 ppm Pbz irrigated plants relative to irrigated control (Fig. S4B). Similarly, Pbz-treatment under deficit irrigation (having elevated ABA levels) recorded even higher protein level of PIP2-7 (compared to Pbz-irrigated) caused by elevated expressions of *SlTIP2* by 4.76- (0.8 ppm) and 5.3-folds (1.6 ppm treated) over the control (Fig. S4C). Elevated expression of PIP2-7 under deficit irrigation in response to Pbz application indicates its active role in stimulating aquaporin (AP) channels’ *de novo* synthesis which facilitates water uptake and management during deficit irrigation. From these observations it is plausible to suggest that increased ABA in Pbz-treated plants under deficit irrigation leads to reduced water loss through stomatal closure^8^ or alternatively manipulated the R/S ratio to manage plant water status through stimulating the activity of AP^37^,^38^.

### Pbz affects central metabolism through enhanced TCA cycle activity under deficit irrigation

**Figure 3:**
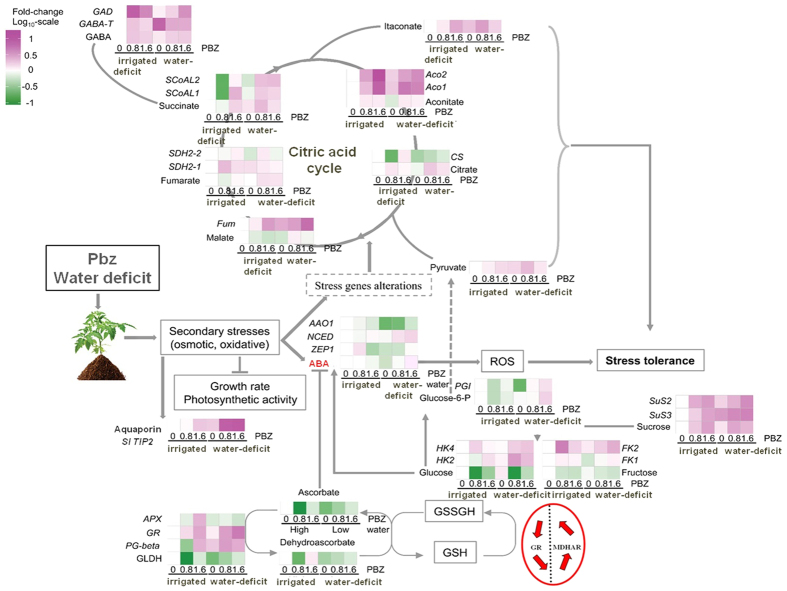
Pbz induced tricarboxylic acid cycle (TCA) adaptations confer tolerance to deficit irrigation. Effects of Pbz application (0, 0.8 and 1.6 ppm) in irrigated (I) and deficit- irrigated (DI) tomato plants on the relative content of the citric/tricarboxylic acid cycle (TCA cycle) intermediates, sugar metabolism and GABA content and the gene expression of enzymes implicated in their metabolism in the leaf tissue of 105-days-old tomato plants as shown in fold change in Log-scale (−1 to +1.5) under irrigated (I) and deficit- irrigated (DI) conditions.

The after effect of a drought episode is to achieve an immediate cellular and biochemical homeostasis. In recent years the field of metabolomics has helped to envisage the physiological picture by enabling a deeper understanding of stress acclimation response of plants^39^. Therefore, to study which metabolites are affected by Pbz application (irrigated/deficit irrigated) leaf metabolism was harnessed by metabolic profiling and a corresponding analysis of expression of key genes involved in TCA cycle was undertaken. A positive correlation could be established between TCA cycle metabolite abundance and corresponding gene expression under water deficit with Pbz concentration (0.8 and 1.6 ppm) compared to irrigated control. For example, a deficit irrigation associated increase in aconitic acid relative content was observed through up-regulation of *Sl Aconitase (SlAco1 and SlAco2*, aconitic acid biosynthesis enzyme) expression (Fig. 3). Similarly a positive correlation was also witnessed between other TCA cycle metabolites such as citrate, succinate and fumarate and up-regulation of genes encoding enzymes of their biosynthesis (Fig. 3).

Pbz (0.8 and 1.6 ppm) application under deficit irrigation was found to increase citrate content 2.18- and 1.64-folds, due to up-regulation of *Sl Citrate synthase (SlCS*, citrate biosynthetic enzyme) by 1.28- and 1.73-folds, respectively compared to Pbz treated irrigated plants (control plants) (Fig. 3). Succinate accumulated (1.63 fold) in Pbz-treated plants under irrigated conditions (1.6 ppm) and expression analysis revealed a 1.66- and 2.01-fold increases in *Sl Succinyl- CoA ligase* (succinate biosynthesis enzyme) *SlSCoAL1* and *SCoAL2* expression, respectively (Fig. 3). Pbz (0.8 ppm) treated deficit irrigated plants showed significantly increased succinate abundance; an outcome of 1.21- and 3.66-fold increases in the expressions of *SCoAL1* and *SCoAL2* expression, respectively (Fig. 3).

The TCA cycle, in addition to producing energy, also meets the onerous demand for carbon skeletons imposed by anabolic processes, such as amino acid synthesis, isoprenoid synthesis, and the control of the carbon to nitrogen (C/N) balance^40^. Oxidative stress (often associated with drought^7^) was shown to have a profound inhibitory effect on the central metabolism in *Arabidopsis thaliana*, including the TCA cycle^40^. In this respect, our findings suggest that Pbz enhances tolerance potential of tomato plants to deficit irrigation through generation of higher energy and carbon skeleton sources for metabolism *via* up-regulating the TCA cycle.

Enhanced GABA production during stressful regimes is a plant’s adaptive response from bypassing the 2-oxoglutarate dehydrogenase (an enzyme using GABA as a substrate)^41^,^42^. Under water deficit, the enzyme 2-oxoglutarate of the TCA cycle is suggested to shift inhibition^43^ of the TCA cycle supported by the activity of the GABA shunt^41^,^42^. Further oxidative stress leads to impaired GABA-shunt thus increasing plant’s susceptibility to oxidative stressors such as drought and heavy metals^37^. Taking into consideration that GABA shunt bypass TCA cycle^41^,^43^ and that the increased level of GABA can suppress TCA cycle intermediates like citrate and malate under C deficiency^44^ we can suggest a shift of C skeletons flow from sugars metabolism to TCA cycle (Fig. 3). The activity of TCA cycle under stress usually decreases^42^. In line with results discussed above we suggest a protective role of Pbz under water limitation. Deficit irrigation (0 DI) induced more GABA accumulation over control irrigated (0 I) plants (Fig. 3). Pbz treated irrigated and deficit irrigated plants exhibited higher accumulation of GABA relative to their respective control plants. This improved GABA accumulation was linked with elevated expression of *glutamate decarboxylase, SlGAD* in Pbz treated irrigated and deficit irrigated plants as compared to their respective control plants. This higher expression of *SlGAD*, an enzyme essential for sustaining glutamate to GABA conversion^43^, suggested a significantly enhanced biosynthesis of GABA. Transcription of GAD is calmodulin dependent; under stress, calcium (Ca^2+^) is known to be released from the mitochondria activating calmodulin and promoting the transcription of genes^45^. Increased tolerance under deficit irrigated conditions is also likely contributed by the Pbz enhanced GABA shunt activity supporting the TCA cycle and contributing to free GABA pools, which can serve as osmolytes^41^,^42^. This view is supported by a significant increase in proline amino acids commonly associated with osmotic adjustment in Pbz treated plants subjected to water deficit stress as compared to deficit irrigated control plants (Table 2). Similarly, glycine and valine also exhibited higher accumulation in 1.6 DI and 0.8 DI plants, respectively. Pbz treated irrigated plants also showed higher accumulation as compared to irrigated control plants. The accumulation of proline, glycine and valine has been demonstrated to be intimately involved in water deficit stress response stabilizing cellular membranes and structures, scavenging free radicals and serving as a precursor for GSH^46^ (in case of glycine).

Furthermore, GABA biosynthesis is linked to polyamine metabolism, which is known to be involved in multiple stress responses including drought in plants^47^. Besides their direct protective role, polyamines (especially spermidine) also regulate various fundamental cellular processes as signaling molecules. It has been shown that abiotic stress tolerance is achieved by the role of polyamines in signaling processes^48^. The conjugation of polyamines (especially spermidine) to photosynthetic complexes leads to enhanced photosynthetic activity under stress conditions^48^. The exogenous spermidine alleviates low temperature injury in *Vigna radiata* by modulating Ascorbate-Glutathione which includes AsA and GSH^49^. In the present study, the 0.8 DI and 1.6 DI plants exhibited higher accumulation of spermidine compared to deficit irrigation (0 DI) control plants (Fig. 2D). Similarly 1.6 I plants also showed higher spermidine levels relative to its irrigated control plants (Fig. 2D). This higher accumulation was further confirmed by the expression analysis of *spermidine synthase (SlSPDS*) (Fig. 2D). So it is plausible to conclude that enhanced photosynthesis and reduced oxidative damage in the 0.8 DI and 1.6 DI plants may be an outcome of increased spermidine *via* modulation of the activity of GSH-ASC cycle.

### Pbz induced sugar metabolism changes

Carbohydrates (Raffinose-family oligosaccharides, disaccharides and fructans) are building blocks of energy that also function as signaling intermediates regulating transcriptional, posttranscriptional and posttranslational processes in plants^18^,^50^,^51^. The outcome of an unfavorable drought stress condition is generation of highly toxic ROS production and subsequent scavenging *via* enzymatic and metabolic antioxidants. In this respect an emerging view relates the abundance of sugars and their associated metabolic enzymes to a plant’s anti-oxidant osmoprotective system^51^,^52^. For example, increased accumulation of proline, anthocyanins and soluble sugars was reported in *Arabidopsis thaliana* leaves imparting high osmoprotection under drought stress^53^. Similarly, paraquat-exposed *Arabidopsis* leaves illustrated adjustments in sugar metabolism as a necessity to survive oxidative stress^54^. Consequently, we analyzed the abundance of glucose, fructose and sucrose metabolites and their corresponding genes to deduce how Pbz treatment integrates deficit irrigation acclimation with osmoprotection. For example, a lower abundance of glucose metabolite levels was observed under control and water deficit plants treated with Pbz (0.8 and 1.6 ppm). This can be attributed to the increased activity of *SlHexokinase* that feeds the TCA cycle for pyruvate *via* conversion of glucose to glucose-6-phosphate (G-6-P) (Fig. 3). Conversely, the levels of endogenous glucose-6-phosphate declined significantly in plants lacking water deficit stress (5.15-fold) whereas under water deficit stress Pbz (1.6 ppm), level of G-6-P increased significantly *via* elevated expression of *Sl phosphoglucomutase (SlPGM,* 7.03-fold compared to control). From the above observations it is proposed that increased sugar levels could maintain the integrity and normal functioning of proteins and membranes under deficit irrigation. While a Pbz-induced increase in TCA metabolism would demand enhanced carbon loading from the glycolysis, which could explain the decreased levels of the hexoses observed in the current study.

### Pbz application enhances anti-oxidant activity *via* alteration of GSH-ASC cycle under deficit irrigation

**Figure 4:**
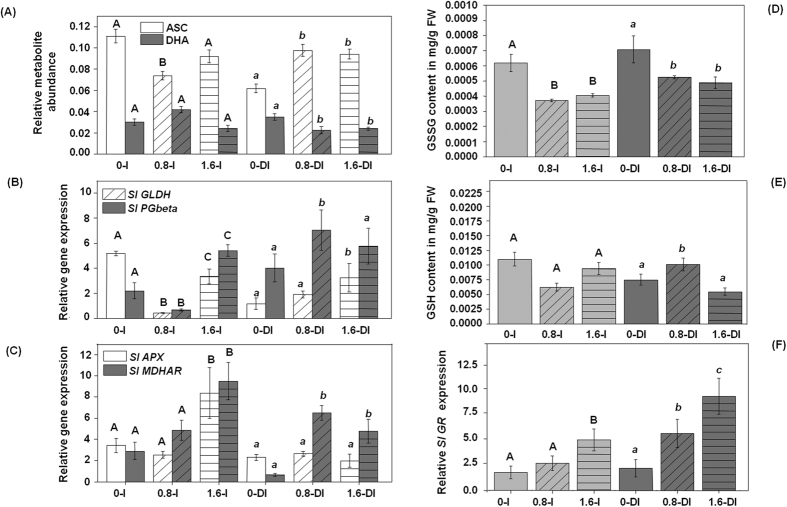
Pbz improved deficit irrigation tolerance by inducing anti-oxidant adaptation *via* modulation of the Glutathione-Ascorbate cycle. Effects of Pbz application (0, 0.8 and 1.6 ppm) in irrigated (I) and deficit-irrigated (DI) tomato plants on the relative content of (**A**) ascorbic acid, ASC and dehydroascorbate, DHA, (**B**,**C**,**F**) expressions analysis of genes related to GSH and ASA biosynthesis (*SlGLDH, SlPGbeta, SlAPX, SlMDHAR* and *SlGR*) and (**D**,**E**) glutathione (GSH and GSSG-ascorbate cycle components) in the leaf tissue of 105-days old tomato plants. Letters (**A**,**B** and a, b) indicate significant differences from the control in irrigated and deficit-irrigated conditions, respectively (one way ANOVA, p < 0.05).

Plants are endowed with low molecular weight non-enzymatic detoxification compounds like ascorbate (ASC) and glutathione (GSH) that may act as redox buffers for quick stress acclimation^55^. The GSH-ASC cycle functions at the heart of cellular redox reactions catalyzing the removal of hydrogen peroxide (H_2_O_2_) produced by the disproportionation of O^2−^ in chloroplasts producing dehydroascorbate (DHA). In this regard, the enzyme DHA reductase constitutes the enzymatic link between cellular ascorbate and GSH pools. Recently GSH has been proposed to be the cellular redox sensor (previously suggested for the ascorbate/DHA redox dyad) due to its high concentration and reduced state^55^,^56^. In the absence of stress plant leaves typically maintain a 20:1 ratio of GSH:GSSG, the deviation from which indicates stress. Moreover, a lower GSH:GSSG ratio under abiotic stress would imply abundance of ascorbate that could inhibit ABA biosynthesis preventing consequent re-acclimation towards normalcy. We analyzed metabolites and expression of genes belonging to this pathway to see how Pbz modulates their activity under control and deficit irrigation conditions. For instance (Fig. 4), a significant increase in ASC (0.8 DI and 1.6 DI) and GSH (0.8 DI) concentration and expression of genes encoding for their respective enzymes *SlGLDH* (l-galactono-1,4-lactone dehydrogenase) and *SlPGbeta* (polygalaturonase-beta) in case of tomato^56^ was observed in Pbz treated water-deficit plants as compared to water-deficit control plants (Fig. 4A,B,E). In essence Pbz application enabled an increase in GSH/GSSG ratio by 2-fold under water-deficit stress over control plants allowing a tight control over the ascorbate-peroxidase pathway and consequently preventing oxidative damage.

These findings are in accordance with previous observations^57^,^58^, reporting higher concentration of ASC in the fruit juice of Pbz-treated citrus lemon, and similarly enhanced GSH level in Pbz-treated *Vigna unguiculata*. In general ASC and GSH are strong antioxidants which scavenge ROS and protect membranes from damage caused incurred by severe drought stress^7^,^55^. Thus, Pbz-induced higher synthesis of ASC and GSH and the maintenance of the GSH/GSSG ratio (data not shown) and GSH-ASC cycle activity ensures sufficient scavenging of ROS generated under water deficit.

### Pbz affects cell wall metabolism, electrolyte leakage and membrane integrity contributing tolerance to water deficit

**Figure 5:**
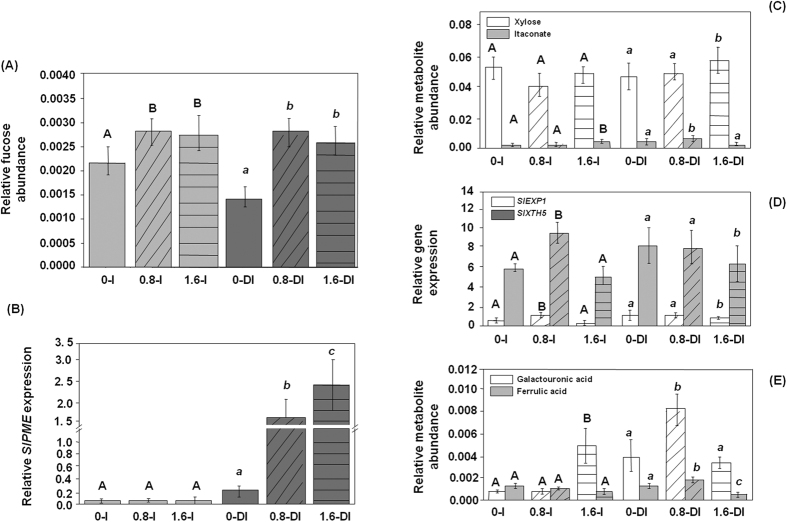
Pbz contributes in tolerance to deficit irrigation by modulating cell wall metabolism. Effects of PBZ application (0, 0.8 and 1.6 ppm) in irrigated (I) and deficit-irrigated (DI) conditions on cell wall metabolites conferring cell wall stability (**A**) fucose, (**C**) xylose and itaconate, (**E**) galactouronic acid and ferrulic acid; and expression analysis of genes related to cell wall organization (**B**,**D**) *SlPME, SlEXP1* and *SlXTH5.* Capital letters (**A**,**B**) indicate significant differences from the PBZ untreated control in the irrigated plants. Small italicized letters (a, b) indicate significant differences from the PBZ untreated control in the deficit-irrigated plants (one way ANOVA, p < 0.05).

Water deficit critically affects cell wall metabolism causing wilting and senescence^7^. The effect of Pbz treatment under water deficit was measured in terms of putative alterations in cell wall metabolites arrangement. The PME (*Pectin methyl esterase*) activity increases cell wall porosity by inducing changes in permeability of the plasmodesmata^59^ and reduces apoplastic pH which in turn activates the local hydrolases XTHs (*Xyloglucan endotransglycosylase/hydrolase*). The XTHs are involved in cell wall biogenesis, cell wall organization, organ abscission and xyloglucan metabolism^60^,^61^,^62^. Expansins are involved in various biological functions like cell wall loosening, cell wall organization, regulation of stomatal movement, unidimensional cell growth, wall disassembly during fruit ripening, abscission and other cell separation events^63^. In the present study, we performed expression analysis of the aforesaid cell wall related genes to discover the putative alterations in cell-wall organization which may contribute tolerance to deficit irrigation. The DI+Pbz treated plants exhibited elevated expressions of *SlPME* as compared to DI control plants (Fig. 5B). However, I+Pbz did not show significant change from relative irrigated control which suggests that Pbz mediated elevation of *SlPME* is dependent on irrigation (Fig. 5B). In case of *SlXTH5*, the DI plants showed higher expression as compared to irrigated control plants which suggest that elevation of *SlXTH5* in DI plants is due to deficit irrigation stress (Fig. 5D). Similarly, *SlEXP1* also exhibited higher expression in DI control plants as compared to irrigated control plants (Fig. 5D). Among secondary sugars, fucose (a hexose deoxy sugar) is a major constituent of plant cell wall surface and plays a key role in cell wall protection^64^. A two fold increase in fucose content was observed in plants during deficit irrigation treated with Pbz (0.8 and 1.6 ppm) when compared to deficit irrigation control plants (Fig. 5A). A similar increase was observed in other cell wall constituents like xylose (1.6 DI plants) and ferrulic acid (0.8 DI plants) in Pbz-treated plants under deficit irrigation stress indicating that Pbz helps in stabilizing and protecting cell wall integrity and permeability during deficit irrigation stress (Fig. 5C,E).

Another indicator of cell membrane integrity is electrical conductance or electrolyte leakage. The onset of drought stress often results in damage to plant tissue that can be quantified using a conductivity meter upon immersion in ion-free water. In principle, cell contents leak at a higher rate due to cell membrane rupture or faulty transmembrane protein pumps that regulate to and fro movement of cell fluids^65^. In the current study, there were no significant difference observed in electrolyte leakage in DI and DI+Pbz treated plant leaves. However, the roots of 1.6 DI plants exhibited significantly reduced electrolyte leakage relative to control DI plants (Fig. S5). This data suggest that Pbz has inhibitory effect on electrolyte leakage in root but no effect in leaf at this stage of plant development. The leaves of both DI and DI+Pbz plants showed reduced electrolyte leakage as compared to irrigated control plants (Fig. S5). The Pbz-induced reductions in EC was in accordance with previous observations^29^.

**Figure 6:**
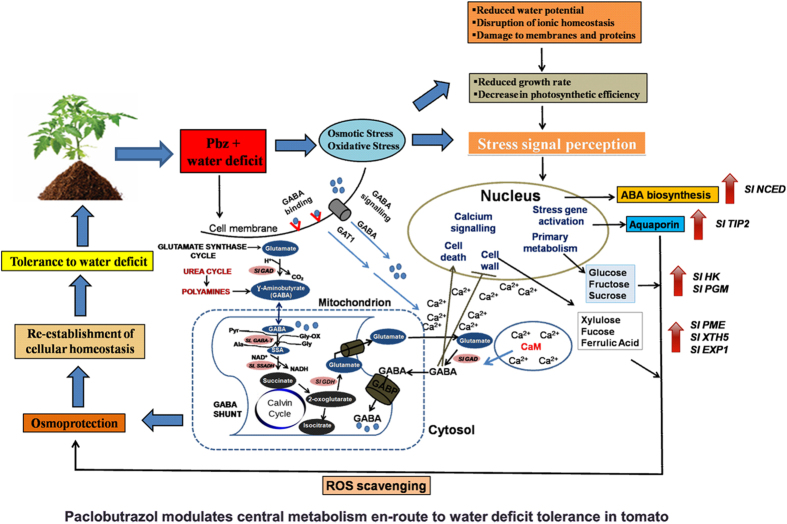
A schematic model describing mechanism of Pbz mediated water deficit tolerance in tomato. Drought stress in plants is accompanied by secondary oxidative or osmotic stress leading to reduction in water potential, disruption of ionic and osmotic homeostasis and damage to proteins and membranes, ultimately resulting in reduced photosynthetic efficiency and overall growth of the plant. Such a signal, perceived in the nucleus leads to activation of stress responsive genes. Similarly, anti-oxidant genes, genes responsible for ABA biosynthesis as well as genes governing primary metabolism (sugar synthesis, glucose, fructose, sucrose) and constituents of cell wall permeability are induced imparting osmoprotection, re-establishment of cellular homeostasis and tolerance to water deficit. Water deficit stress when applied in conjunction with Pbz engenders modulation of central metabolism through enhanced TCA cycle activity and regulation of gene expression associated with GABA shunt signaling. Generally, external stimuli like drought stress or deficit irrigation described here lead to increase in endogenous GABA levels permitting its adherence to cell-surface binding sites, enabling an interim increase in Ca^2+^ pools and its import into cells through high affinity GABA transporters (e.g., GAT1^74^). Considerably, GAD is activated through a Ca^2+^/CaM complex^45^,^75^. Thereafter, increased intracellular GABA activates various signaling cascades and genes of primary metabolism (*SlHK* and *SlPGM*) while inhibiting some, like genes responsible for cell wall-modifications. Additionally, subject to outside environment, a substantial portion of cytosolic GABA makes way into mitochondria *via* the GABA permease, At GABP^76^, for catabolism by GABA-T and SSADH, producing succinate for feeding into the Calvin Cycle. Enzyme represent oval grey boxes, their reactions represented by black arrows. Brown lines indicate a regulatory effect. Blue spheres denote GABA, red crescents denote GABA receptors. Abbreviations: Pbz, Paclobutrazol; GABA, γ-aminobutric acid; GAT1, GABA transporter 1 *SlNCED*, 9-cis-epoxy-carotenoid dioxygenase; *SlTIP2*, tonoplast intrinsic protein 2; *SlHK*, hexokinase; *SlPGM*, phosphoglucomutase; *SlPME*, Pectin methyl esterase; *SlXTH5*, Xyloglucan endotransglycosylase; *SlEXP1*, expansin1; *SlGDH*, Glutamate dehydrogenase; CaM, calmodulin; GAD, glutamate decarboxylase; GABA-T, GABA transaminase; SSADH, succinic semialdehyde dehydrogenase; respectively.

The present study has provided detailed insights into Pbz induced water deficit irrigation tolerance mechanism in tomato plants (Fig. 6). Pbz modified morphological parameters causing reduced height and increased stem diameter thus leading to smaller plants with relatively better performance under water deficit stress. Pbz increased leaf thickness, chlorophyll content, stomatal conductance and photosynthesis. Even though the total photosynthates were less because of the significantly reduced leaf area, the growth condition of Pbz treated plants was considerably better than non Pbz-treated plants. The RGR of tomato plants was higher after Pbz application during water deficit stress. Moreover, Pbz increased both dry weight and area of basal root to primary root ratio, that made roots of Pbz treated plants more accessible to water and nutrients in the upper soil. The increased SRA of the first two orders’ root and decreased SRA of the third order root were not only able to improve water and nutrient uptake efficiency but also transport capability, that ensured plants had enough resource access (unpublished results). On the other hand, the increased NPQ in Pbz-treated water-deficit plants implied higher ability in scavenging excessive photo damage through heat dissipation. At metabolomics and expression level, higher accumulation of intermediates of the TCA cycle and expression of key enzymes of TCA, signify higher TCA cycle activity in mitochondria, providing more energy and carbon skeletons (Fig. 6). GABA shunt reduced the risks of excess reductants coming from up-regulated TCA cycle enabling plants better acclimation to drought stress (Fig. 6). Taken together the higher content of GSH and ASC, Pbz treated water-deficit tomato plants were better suited for defense against antioxidants and maintaining better growth and viability under drought stress. The higher content of fucose, itaconate, proline and glycine and less electrolyte leakage in Pbz treated plants under water deficit ensures higher membrane stability under drought stress. Above all, even though the plants were grown in sandy loam, Pbz still increased plant’s tolerance to deficit irrigation at morphological, metabolic and molecular level. This study thus provides us with an impending tool to generate drought tolerant plants *via* Pbz application in an eco-friendly way.

## Materials and Methods

### Plant material and growth conditions

Seeds of *Solanum lycopersicum* L. var. Mose were supplied by Syngenta company (Syngenta Corp. Ltd.) (Zeraim Gedera), Kibutz Revadim, Israel. After surface sterilization with 0.4% sodium hypochlorite for 15 min, seeds were sown in pots (18 L capacity, 308 mm height, 260 mm in diameter), filled with sterilized sandy loam soil (24 kg pot^−1^) consisting of 51.4% silt, 8.8% clay and 39.8% sand. Water was applied automatically through surface drip irrigation once a day based on calculated plant evaporative demand. The experiment was conducted in a multi-span greenhouse at the Sede Boqer campus (30°52′08.04″N and 34°47′33″E) of Ben-Gurion University, Israel from 6^th^ November 2011 to 20^th^ February 2012. Growth conditions inside the greenhouse were as follows: max/min temperature 25/17 °C (day/night), mean relative humidity 70%, and photosynthetic photon flux density (PPFD) 800 μmol m^−2^ s^−1^ (photoperiod, 14 h). In the greenhouse, 150 plants, one plant/per pot, were arranged in a randomized block design. Fifty-six days old plants were divided into two groups, with first group consisting of 75 plants (25 plants each for control 0, 0.8 and 1.6 ppm Pbz condition) constituting the control group subjected to normal irrigation conditions (i.e. 100% of the evaporative demand), while the second group with same conditions and number of plants were subjected to deficit-irrigation condition (70% of evaporative demand) for a period of 50 days. Daily water balance, generating transpiration data for each pot, was calculated based on T = I − D − ΔM where I is the irrigation, D is the drainage and ΔM indicates change in the soil water mass measured with weighing the pots twice a week^66^. This algorithm of irrigation yielded an average cumulative amount of 14.4 L for the well-watered plants and 10.2 L for the deficit plants.

Flowers were removed at regular intervals to avoid effects of reproductive organ development on the experimental set up and water status of the plants under study. The 105 days old plants were harvested to determine the effects of Pbz on irrigated and deficit irrigated regimes at the metabolomic and molecular level in plants. In one independent experiment, 24–25 plants were used for each treatment. Three independent experiments were performed. For each biological replicates three technical replicates were performed.

### Pbz application

The soil drench method^67^ was used for the application of three concentrations of Pbz (0, 0.8 and 1.6 ppm) directly to the seeds. The Pbz solution was applied circumferentially close to the individual seed in each pot.

### Chemicals and reagents

All the chemicals used in this study were purchased from Israel. Paclobutrazol (Pbz) ([2RS, 3RS]-1-[4-chlorophenyl]-4,4-dimethyl-2-[1,2,4-triazol-1-yl] pentan- 3-ol) used in this study was provided by Syngenta Corp. Ltd. (Zeraim Gedera), Kibutz Revadim, Israel. An Enzyme-Linked Immunosorbent Assay kit (ELISA PDK 09347/0096) for the estimation of ABA was purchased from Agdia-Biofords, France. An anti-PIP2-7 (plasma membrane aquaporin C-terminal) antibody was purchased from Agrisera AB, Vännäs, Sweden.

### Morphological parameters

Plant height, number of leaves, leaf area (LA), and stem thickness were measured twice a week. Root surface area (RA) was determined at the 80^th^ day after planting (DAP) by separately scanning a subset of basal roots and the primary roots with laterals using Epson Expression 10000XL with a transparency unit. Specific root area (SRA) was determined using the software WinRhizo 2005c (Régent Instruments Inc., Québec, QC, Canada). The following formula was used to determine the total root surface area (RA):


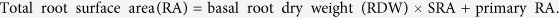


### Biomass

Relative growth rate (RGR) was determined on dry weight basis of harvested plants. Shoot dry weight (ShDW) and root dry weight (RDW) were measured using a fine weighing scale (EI-i series, A&D Company Limited).

### Physiological parameters

Photosynthesis, stomatal conductance, NPQ and electron transport rate (ETR) were measured with a portable photosynthesis system twice a week (LI-6400XT; LI-COR, Lincoln, NE, USA). In brief, green leaves were enclosed in the IRGA under a light intensity of 700 PPFD, 400 μ mol mol^−1^ CO_2_ at 25 °C leaf temperature at relative humidity between 40 and 55%.

Chlorophyll (Chl) content was determined with CCM-200 plus portable fluorometer (Opti-Sciences, USA). From a pool of plants for each treatment (25 plants/treatment) Chl content index was calculated. Drainage collection was determined on weekly basis. Water-use efficiency (WUE) was calculated using a formula described in ref. 68. All the parameters were measured on the third leaf from the plant apex at same time of the day (11 am) to remove circadian effects.

### Metabolite profiling

Metabolite profiling was performed with a Thermo Scientific DSQ II GC/MS as exactly described in Lisec *et al*.^69^ (please see also supporting information). Metabolite extraction, analysis and identification were exactly as described^69^.

### RNA isolation, cDNA preparation and quantitative real-time PCR

Total RNA was extracted from liquid N_2_ snap-frozen tomato leaves using TRI reagent (Sigma-Aldrich, Israel) as per the manufacturer’s instructions. qPCR was performed with an ABI PRISM 7500 Sequence Detection System (SDS) (Applied Biosystems, Life Technologies, CA, USA). Each reaction contained 5 μl PerfeCTa^®^ SYBR^®^ Green Fast Mix^®^ (Quanta Biosciences), 40 ng cDNA and 300 nM of gene-specific primer in a final volume of 10 μl. PCR amplifications were performed using the following conditions: 95 °C for 30 s, 40 cycles of 95 °C for 5 s (denaturation) and 60 °C for 35 s (annealing/extension). Data was analyzed using the SDS 1.3.1 software (Applied Biosystems). The expression of 32 genes-encoding enzymes involved in the tricarboxylic acid (TCA) cycle, sugars, ascorbic acid (ASC), the glutathione (GSH)-ASC cycle, GABA metabolism, cell wall stability, ABA and spermidine metabolism were analyzed (Table S1). Most of the primers used for the qPCR analysis were taken from published data, while some primers of the tomato genes were designed using their *Arabidopsis* orthlologs for a homology search against the EST databank of *S. lycopersicum* available at www.plantgdb.org and www.pubmed.com. Gene specific primer pairs for each gene were designed as described^70^. qPCR data was analyzed using SDS 1.3.1 software (Applied Biosystems) and relative quantification values for each target gene was calculated by the 2^−∆∆CT^ method^71^, using *Actin Tom 41*(U60480)as a reference gene. Gene expression was normalized to the expression level of irrigated plants without Pbz (before water deficit stress started), which was assigned a value of 1. The specificity of the all the primers were checked using dissociation curve analysis at the end of each run. All reactions were performed in triplicates. All RT-qPCR experiments were repeated three times using cDNAs prepared from three independent biological samples of tomato leaf tissues representing conditions tested.

### ELISA competitive assay for ABA quantification

The ABA quantification was performed with the ELISA kit as per the manufacturer’s instructions. Standards and samples were run in duplicates and the concentration of ABA was calculated using the formulae provided with the kit.

### Western blotting of aquaporin (water channel protein)

Expression of AP was determined using plasma membrane intrinsic proteins (PIP2-7) antibody as described previously^36^.

### Measurement of stress indices

Total GSH (reduced) and GSSG (oxidized) content was determined with UPLC-Q-TOF-MS (Waters Corp., Manchester, UK)^72^. The relative content of amino acids implicated in water deficit tolerance; glycine (Gly), glutamine (Gln), glutamic acid (Glu), proline (Pro), leucine (Leu), serine (Ser), tryptophan (Trp) and valine (Val) was determined with a Thermo Scientific DSQ II GC/MS using a Factor Four Capillary VF-5ms column^69^. Electrolyte conductance (EC) was determined as described previously^10^,^11^.

### Statistical analysis

Unless otherwise stated, all the experiments were repeated three times, and within each experiment, treatments (0, 0.8, 1.6 ppm Pbz, irrigated and deficit-irrigated) were replicated five times, with each replication comprising of 24–25 pooled plants. A one-way analysis of variance (ANOVA) was carried out using p < 0.05 as a measure of significance. All statistical calculations were performed using MeV 4.9 software^73^. The results of metabolite analysis were visualized in a heat-map view using “ggplot2” (H. Wickham. ggplot2: Elegant Graphics for Data Analysis. Springer-Verlag New York, 2009) package for R-project (R Development Core Team (2008). R: A language and environment for statistical computing. R Foundation for Statistical Computing, Vienna, Austria. ISBN 3-900051-07-0, URL http://www.R-project.org.).

## Additional Information

**How to cite this article:** Pal, S. *et al*. Paclobutrazol induces tolerance in tomato to deficit irrigation through diversified effects on plant morphology, physiology and metabolism. *Sci. Rep.* **6**, 39321; doi: 10.1038/srep39321 (2016).

**Publisher's note:** Springer Nature remains neutral with regard to jurisdictional claims in published maps and institutional affiliations.

## Supplementary Material

Supplementary Information

## Figures and Tables

**Table 1:** Effects of Pbz application (0, 0.8 and 1.6 ppm) in irrigated (I) and deficit-irrigated (DI) states on the physiological parameters; photosynthesis, stomatal conductance, electron transport rate (ETR), non-photochemical quenching (NPQ) and water use efficiency (WUE) of tomato leaves.

	Irrigated (I)			Deficit-irrigated (DI drought)		
	PBZ, [ppm]			PBZ, [ppm]		
	0	0.8	1.6	0	0.8	1.6
**Photosynthesis**, **[μM(CO**_**2**_**) m**^**−2**^ **s**^**−1**^]	13.35 ± 1.58 ^A^	16.02 ± 1.33^B^	15.08 ± 1.53 ^A^	10.15 ± 1.28^*a*^	14.39 ± 2.03^*b*^	13.6 ± 1.23^*b*^
**Stomatal conductance**, **[mol (H**_**2**_**O) m**^**−2**^ **s**^**−1**^]	0.53 ± 0.06 ^A^	0.59 ± 0.06 ^A^	0.6 ± 0.06 ^A^	0.4 ± 0.05^*a*^	0.52 ± 0.05^*b*^	0.53 ± 0.05^*b*^
**ETR**, **[μM m**^**−2**^ **s**^**−1**^]	159.83 ± 6.95 ^A^	176.65 ± 3.12^B^	179.83 ± 5.37^B^	145.76 ± 8.36^*a*^	166.5 ± 5.08^*b*^	164.62 ± 4.6^*b*^
**NPQ**	0.8 ± 0.1 ^A^	0.64 ± 0.12^B^	0.62 ± 0.14^B^	0.81 ± 0.13^*a*^	0.87 ± 0.16^*a*^	0.82 ± 0.14^*a*^
**WUE**, **[μM(CO**_**2**_**)/mM(H**_**2**_**O)]**	2.28 ± 0.28 ^A^	3.17 ± 0.21^B^	3.87 ± 0.26 ^C^	1.66 ± 0.26^*a*^	1.96 ± 0.29^*b*^	1.68 ± 0.2^*a*^

Capital letters (A,B) indicate significant differences from the untreated control in irrigated state, while small italicized letters (a,b) indicate significant differences from the untreated control in the DI (one way ANOVA, p < 0.05).

**Table 2:** Effects of Pbz application (0, 0.8 and 1.6 ppm) in irrigated (I) and deficit-irrigated (DI) states on the physiological parameters on the relative amino acid content of leaf tissue of tomato plants.

	Glycine	Glutamine	Glutamic acid	Proline	Leucine	Serine	Tryptophan	Valine
**0**, **I**	0.004953 ± 0.0013^A^	0.00256 ± 0.0010^A^	0.036936 ± 0.0155^A^	0.001077 ± 0.0004^A^	0.00136 ± 0.0005^A^	0.010116 ± 0.003^A^	0.004706 ± 0.0002^A^	0.00796 ± 0.0029^A^
**0.8**, **I**	0.002763 ± 0.0010^B^	0.00331 ± 0.0012^A^	0.107957 ± 0.0536^B^	0.000365 ± 0.0001^B^	0.002436 ± 0.0007^B^	0.022497 ± 0.0072^B^	0.014125 ± 0.0048^B^	0.01117 ± 0.0027^A^
**1.6**, **I**	0.003407 ± 0.0015^A^	0.004363 ± 0.0018^B^	0.056395 ± 0.0198^A^	0.000642 ± 0.0002^B^	0.003696 ± 0.0010^C^	0.037733 ± 0.0146^C^	0.013534 ± 0.0059^B^	0.02012 ± 0.0064^B^
**0-DI**	0.004261 ± 0.0018^*a*^	0.00601 ± 0.0026^*a*^	0.036511 ± 0.0152^*a*^	0.000504 ± 8E-05^*a*^	0.001397 ± 0.0004^*a*^	0.010081 ± 0.0019^*a*^	0.001903 ± 0.0004^*a*^	0.008373 ± 0.0037^*a*^
**0.8-DI**	0.002456 ± 0.0008^b^	0.00623 ± 0.0028^*a*^	0.030391 ± 0.0103^*a*^	0.000892 ± 0.0002^*b*^	0.00214 ± 0.0009^*b*^	0.026371 ± 0.0096^*b*^	0.00047 ± 9.57E-05^*b*^	0.01017 ± 0.0044^*a*^
**1.6-DI**	0.005737 ± 0.0024^*a*^	0.001754 ± 0.0007^*b*^	0.064254 ± 0.0257^*b*^	0.000804 ± 0.0002^*b*^	0.000897 ± 0.0002^*c*^	0.019323 ± 0.0061^*c*^	0.00022 ± 6.79E-05^*b*^	0.005654 ± 0.0018^*b*^

Capital letters (A,B) indicate significant differences from the untreated control in irrigated state, while small italicized letters (a,b) indicate significant differences from the untreated control in the DI (one way ANOVA, p < 0.05).

## References

[b1] Loyola J., Verdugo I., González E., Casaretto J. A. & Ruiz-Lara S. Plastidic isoprenoid biosynthesis in tomato: physiological and molecular analysis in genotypes resistant and sensitive to drought stress. Plant Biol. (Stuttg) 14, 149–156 (2012). 21974688 10.1111/j.1438-8677.2011.00465.x

[b2] FAOSTAT 2014. Area harvested and Yield - Tomatoes 2012. Rome, Italy: Food and Agricultural Organization of the United Nations. Availabe at: http://faostat.fao.org/site/567/DesktopDefault.aspx?PageID=567 (Accessed: 28^th^ October 2014).

[b3] Fereres E. & Soriano M. A. Deficit irrigation for reducing agricultural water use. J. Exp. Bot. 58, 147–59 (2007). 17088360 10.1093/jxb/erl165

[b4] Tester M. & Langridge P. Breeding technologies to increase crop production in a changing world. Science 327, 818–822 (2010). 20150489 10.1126/science.1183700

[b5] Chai Q. . Regulated deficit irrigation for crop production under drought stress. A review. Agron. Sustain. Dev. 36, 3 (2016).

[b6] Skirycz A. & Inzé D. More from less: plant growth under limited water. Curr. Opin. Biotechnol. 21, 197–203 (2010). 20363612 10.1016/j.copbio.2010.03.002

[b7] Miller G., Suzuki N., Ciftci-Yilmaz S. & Mittle R. Reactive oxygen species homeostasis and signalling during drought and salinity stresses. Plant Cell Environ. 33, 453–467 (2010). 19712065 10.1111/j.1365-3040.2009.02041.x

[b8] Shatil-Cohen A., Attia Z. & Moshelion M. Bundle-sheath cell regulation of xylem-mesophyll water transport via aquaporins under drought stress: a target of xylem-borne ABA? Plant J. 67, 72–80 (2011). 21401747 10.1111/j.1365-313X.2011.04576.x

[b9] Pinheiro H. A. . Drought tolerance is associated with rooting depth and stomatal control of water use in clones of *Coffea canephora*. Ann. Bot. 96, 101–108 (2005). 15888500 10.1093/aob/mci154 PMC4246813

[b10] Choudhary S. P. Yu J. Q., Yamaguchi-Shinozaki K., Shinozaki K. & Tran L. S. P. Benefits of brassinosteroid crosstalk. Trends Plant Sci. 17, 594–605 (2012a). 22738940 10.1016/j.tplants.2012.05.012

[b11] Choudhary S. P. . Chromium stress mitigation by polyamine-brassinosteroid application involves phytohormonal and physiological strategies in *Raphanus sativus* L. PLoS One 7, e33210 (2012b). 22479371 10.1371/journal.pone.0033210 PMC3315560

[b12] Chen Z. . Expression analysis of abscisic acid (ABA) and metabolic signalling factors in developing endosperm and embryo of barley. J. Cereal Sci. 58, 255–262 (2013). 24748715 10.1016/j.jcs.2013.06.009 PMC3990443

[b13] Miura K. . SIZ1 deficiency causes reduced stomatal aperture and enhanced drought tolerance via controlling salicylic acid‐induced accumulation of reactive oxygen species in *Arabidopsis*. Plant J. 73, 91–104 (2013). 22963672 10.1111/tpj.12014

[b14] Park H. C., Cha J. Y. & Yun D. J. Roles of YUCCAs in auxin biosynthesis and drought stress responses in plants. Plant Signal. Behav. 8, e24495 (2013). 23603963 10.4161/psb.24495 PMC3907447

[b15] Zhang C. & Huang Z. Effects of endogenous abscisic acid, jasmonic acid, polyamines, and polyamine oxidase activity in tomato seedlings under drought stress. Sci. Hortic. 159, 172–177 (2013).

[b16] Pérez-Alfocea F., Ghanem M. E., Gómez-Cadenas A. & Dodd I. C. Omics of root-to-shoot signaling under salt stress and water deficit. OMICS 15, 893–901(2011). 22136663 10.1089/omi.2011.0092

[b17] Witt S. . Metabolic and phenotypic responses of greenhouse-grown maize hybrids to experimentally controlled drought stress. Mol. Plant. 5, 401–417 (2012). 22180467 10.1093/mp/ssr102

[b18] Seki M., Umezawa T., Urano K. & Shinozaki K. Regulatory metabolic networks in drought stress responses. Curr. Opin. Plant Biol. 10, 296–302 (2007). 17468040 10.1016/j.pbi.2007.04.014

[b19] Matsui A. . *Arabidopsis* transcriptome analysis under drought, cold, high-salinity and ABA treatment conditions using a tiling array. Plant Cell Physiol. 49, 1135–1149 (2008). 18625610 10.1093/pcp/pcn101

[b20] Mittler R. & Blumwald E. Genetic engineering for modern agriculture: challenges and perspectives. Annu. Rev. Plant Biol. 61, 443–462 (2010). 20192746 10.1146/annurev-arplant-042809-112116

[b21] Lawlor D. W. Genetic engineering to improve plant performance under drought: physiological evaluation of achievements, limitations, and possibilities. J. Exp. Bot. 64, 83–108 (2013). 23162116 10.1093/jxb/ers326

[b22] Peleg Z. & Blumwald E. Hormone balance and abiotic stress tolerance in crop plants. Curr. Opin. Plant Biol. 14, 290–295 (2011). 21377404 10.1016/j.pbi.2011.02.001

[b23] Srivastav M., Kishor A., Dahuja A. & Sharma R. R. Effect of paclobutrazol and salinity on ion leakage, proline content and activities of antioxidant enzymes in mango (*Mangifera indica* L.). Sci. Hortic. 125, 785–788 (2010).

[b24] Shahrokhi M., Tehranifar A., Hadizadeh H. & Selahvarzi Y. Effect of drought dtress and daclobutrazol- dreated deeds on dhysiological desponse of *Festuca arundinacea* L. Master and *Lolium perenne* L. Barrage. J. Biol. Environ. Sci. 5, 77–85 (2011).

[b25] Upreti K. K. . Hormonal changes in response to paclobutrazol induced early flowering in mango cv. Totapuri. Sci. Hortic. 150, 414–418 (2013).

[b26] Baninasab B. & Ghobadi C. Influence of paclobutrazol and application methods on high-temperature stress injury in cucumber seedlings. J. Plant Growth Regul. 30, 213–219 (2011).

[b27] Berova M. & Zlatev Z. Physiological response of paclobutrazol-treated Triticale plants to water stress. Biol. Planta. 46, 133–136 (2003).

[b28] Oliver M. J., Tuba Z. & Mishler B. D. The evolution of vegetative desiccation tolerance in land plants. Plant Ecol. 151, 85–100 (2000).

[b29] Gopi R. . Differential effects of hexaconazole and paclobutrazol on biomass, electrolyte leakage, lipid peroxidation and antioxidant potential of *Daucus carota* L. Colloids and surfaces. Biointerfaces 60, 180–186 (2007). 17644352 10.1016/j.colsurfb.2007.06.003

[b30] Bayat S. & Sepehri A. Paclobutrazol and salicylic acid application ameliorates the negative effect of water stress on growth and yield of maize plants. J. Res. Agri. Sci. 8, 127–139 (2012).

[b31] Berova M. & Zlatko Z. Physiological response and yield of paclobutrazol treated tomato plants (*Lycopersicon esculentum* Mill.). Plant Growth Regul. 30.2, 117–123 (2000).

[b32] Nivedithadevi D., Somasundaram R. & Pannerselvam R. Effect of abscisic acid, paclobutrazol and salicylic acid on the growth and pigment variation in *Solanum trilobatum* (l). Int. J. Drug Dev. Res. 4, 236–246 (2012).

[b33] Lambrev P. H., Miloslavina Y., Jahns P. & Holzwarth A. R. On the relationship between non-photochemical quenching and photoprotection of Photosystem II. Biochim. Biophys. Acta 1817, 760–9 (2012). 22342615 10.1016/j.bbabio.2012.02.002

[b34] Abdul J. C. . Paclobutrazol enhances photosynthesis and ajmalicine production in *Catharanthus roseus*. Process Biochem. 42, 1566–1570 (2007).

[b35] Rivero R. M., Shulaev V. & Blumwald E. Cytokinin-dependent photorespiration and the protection of photosynthesis during water deficit. Plant Physiol. 150, 1530–1540 (2009). 19411371 10.1104/pp.109.139378 PMC2705023

[b36] Sade N. . Improving plant stress tolerance and yield production: is the tonoplast aquaporin SlTIP2; 2 a key to isohydric to anisohydric conversion? New Phytol. 181, 651–661(2009). 19054338 10.1111/j.1469-8137.2008.02689.x

[b37] Parent B. . Drought and abscisic acid effects on aquaporin content translate into changes in hydraulic conductivity and leaf growth rate: a trans-scale approach. Plant Physiol. 149, 2000–2012 (2009). 19211703 10.1104/pp.108.130682 PMC2663730

[b38] Mahdieh M. & Mostajeran A. Abscisic acid regulates root hydraulic conductance via aquaporin expression modulation in *Nicotiana tabacum*. J Plant Physiol. 166, 1993–2003 (2009). 19576659 10.1016/j.jplph.2009.06.001

[b39] Krasensky J. & Jonak C. Drought, salt, and temperature stress-induced metabolic rearrangements and regulatory networks. J. Exp. Bot. 63, 1593–608 (2012). 22291134 10.1093/jxb/err460 PMC4359903

[b40] Baxter C. J. . The metabolic response of heterotrophic *Arabidopsis* cells to oxidative stress. Plant Physiol. 143, 312–325 (2007). 17122072 10.1104/pp.106.090431 PMC1761969

[b41] Fait A. . Highway or byway: the metabolic role of the GABA shunt in plants. Trends Plant Sci. 13, 14–9 (2008). 18155636 10.1016/j.tplants.2007.10.005

[b42] Sweetlove L. J. . Not just a circle: flux modes in the plant TCA cycle. Trends Plant Sci. 15, 462–470 (2010). 20554469 10.1016/j.tplants.2010.05.006

[b43] Bouche N., Fait A., Zik M. & Fromm H. The root-specific glutamate decarboxylase (GAD1) is essential for sustaining GABA levels in *Arabidopsis*. Plant Mol. Biol. 55, 315–325 (2004). 15604684 10.1007/s11103-004-0650-z

[b44] Batushansky A. . Combined transcriptomics and metabolomics of *Arabidopsis thaliana* seedlings exposed to exogenous GABA suggest its role in plants is predominantly metabolic. Mol. Plant. 7, 1065–1068 (2014). 24553152 10.1093/mp/ssu017

[b45] Baum G. . Calmodulin binding to glutamate decarboxylase is required for regulation of glutamate and GABA metabolism and normal development in plants. EMBO J. 15, 2988–2996 (1996). 8670800 PMC450240

[b46] Yang S. L., Lan S. S. & Gong M. Hydrogen peroxide-induced proline and metabolic pathway of its accumulation in maize seedlings. J. Plant Physiol. 166, 1694–1699 (2009). 19446917 10.1016/j.jplph.2009.04.006

[b47] Bitrián M. . Polyamines under abiotic stress: metabolic crossroads and hormonal crosstalks in plants. Metabolites 2, 516–528 (2012). 24957645 10.3390/metabo2030516 PMC3901213

[b48] Pál M., Szalai G. & Janda T. Speculation: Polyamines are important in abiotic stress signaling. Plant Sci. 237, 16–23 (2015). 26089148 10.1016/j.plantsci.2015.05.003

[b49] Nahar K., Hasanuzzaman M., Alam M. M. & Fujita M. Exogenous spermidine alleviates low temperature injury in mung bean (*Vigna radiata* L.) seedlings by sodulating ascorbate-glutathione and glyoxalase pathway. Int. J. Mol. Sci. 16, 30117–30132 (2015). 26694373 10.3390/ijms161226220 PMC4691163

[b50] Ruan Y. L. Sucrose metabolism: gateway to diverse carbon use and sugar signaling. Annu. Rev. Plant Biol. 65, 33–67 (2014). 24579990 10.1146/annurev-arplant-050213-040251

[b51] Liu Y. H., Offler C. E. & Ruan Y. L. Regulation of fruit and seed response to heat and drought by sugars as nutrients and signals. Front. Plant Sci. 4, 282 (2013). 23914195 10.3389/fpls.2013.00282 PMC3729977

[b52] Keunen E., Peshev D., Vangronsveld J., Van den Ende W. & Cuypers A. Plant sugars are crucial players in the oxidative challenge during abiotic stress: extending the traditional concept. Plant Cell Environ. 36, 1242–1255 (2013). 23305614 10.1111/pce.12061

[b53] Sperdouli I. & Moustakas M. Interaction of proline, sugars, and anthocyanins during photosynthetic acclimation of *Arabidopsis thaliana* to drought stress. J. Plant Physiol. 169, 577–85 (2012). 22305050 10.1016/j.jplph.2011.12.015

[b54] Scarpeci T. E., Zanor M. I., Carrillo N., Mueller-Roeber B. & Valle E. M. Generation of superoxide anion in chloroplasts of *Arabidopsis thaliana* during active photosynthesis: a focus on rapidly induced genes. Plant Mol. Biol. 66, 361–378 (2008). 18158584 10.1007/s11103-007-9274-4 PMC2758387

[b55] Foyer C. H. & Noctor G. Redox signaling in plants. Antioxid. Redox Sign. 18, 2087–2090 (2013). 10.1089/ars.2013.5278 23442120

[b56] Gallie D. R. The role of L-ascorbic acid recycling in responding to environmental stress and in promoting plant growth. J. Exp. Bot. 64, 433–443 (2013). 23162122 10.1093/jxb/ers330

[b57] Jain S. K., Singh R. & Misra K. K. Effect of paclobutrazol on growth, yield and fruit quality of lemon (*Citrus limon*). Indian J. Agri. Sci. 72, 488–490 (2002).

[b58] Manivannan P. . Protection of *Vigna unguiculata* (L.) Walp. plants from salt stress by paclobutrazol. Colloids Surf B: Biointerfaces 61, 315–318 (2008). 17961996 10.1016/j.colsurfb.2007.09.007

[b59] Chen M. H., Sheng J., Hind G., Handa A. K. & Citovsky V. Interaction between the tobacco mosaic virus movement protein and host cell pectin methylesterases is required for viral cell‐to‐cell movement. EMBO J. 19, 913–920 (2000). 10698933 10.1093/emboj/19.5.913 PMC305631

[b60] Rose J. K., Braam J., Fry S. C. & Nishitani K. The XTH family of enzymes involved in xyloglucan endotransglucosylation and endohydrolysis: current perspectives and a new unifying nomenclature. Plant Cell Physiol. 43, 1421–1435 (2002). 12514239 10.1093/pcp/pcf171

[b61] Singh A. P., Tripathi S. K., Nath P. & Sane A. P. Petal abscission in rose is associated with the differential expression of two ethylene-responsive xyloglucan endotransglucosylase/hydrolase genes, RbXTH1 and RbXTH2. J. Exp. Bot. 62, 5091–5103 (2011). 21765161 10.1093/jxb/err209 PMC3193013

[b62] Singh A. P. . Differential expression of several xyloglucan endotransglucosylase/hydrolase genes regulates flower opening and petal abscission in roses. AoB Plants 5, plt030 (2013).

[b63] Cosgrove D. J. New genes and new biological roles for expansins. Curr. Opin. Plant Biol. 3, 73–78 (2000). 10679451 10.1016/s1369-5266(99)00039-4

[b64] Reiter W. D., Chapple C. C. & Somerville C. R. Altered growth and cell walls in a fucose-deficient mutant of *Arabidopsis*. Science 261, 1032–1035 (1993). 17739625 10.1126/science.261.5124.1032

[b65] Whitlow T. H., Bassuk N. L., Ranney T. G. & Reichert D. L. An improved method for using electrolyte leakage to assess membrane competence in plant tissues. Plant Physiol. 98, 198–205 (1992). 16668614 10.1104/pp.98.1.198 PMC1080169

[b66] Ityel E., Lazarovitch N., Silberbush M. & Ben-Gal A. An artificial capillary barrier to improve root-zone conditions for horticultural crops: Response of pepper plants to matric head and irrigation water salinity. Agri. Water Manage. 105, 13–20 (2012).

[b67] Xu G., Luo R. & Yao Y. Paclobutrazol improved the reproductive growth and the quality of seed oil of *Jatropha curcas*. J Plant Growth Regul. 32, 875–883 (2013).

[b68] Yang L., Qu H., Zhang Y. & Li F. Effects of partial root-zone irrigation on physiology, fruit yield and quality and water use efficiency of tomato under different calcium levels. Agri. Water Manage. 104, 89–94 (2012).

[b69] Lisec J. . Gas chromatography mass spectrometry–based metabolite profiling in plants. Nat. Protoc. 1, 387–396 (2006). 17406261 10.1038/nprot.2006.59

[b70] Kant S., Kant P., Raveh E. & Barak S. Evidence that differential gene expression between the halophyte, *Thellungiella halophila*, and *Arabidopsis thaliana* is responsible for higher levels of the compatible osmolyte proline and tight control of Na^+^ uptake in *T. halophila*. Plant Cell Environ. 29, 1220–1234 (2006). 17080945 10.1111/j.1365-3040.2006.01502.x

[b71] Schmittgen T. D. & Livak K. J. Analyzing real-time PCR data by the comparative C(T) method. Nat. Protoc. 3, 1101–1108 (2008). 18546601 10.1038/nprot.2008.73

[b72] Brychkova G., Yarmolinsky D., Fluhr R. & Sagi M. The determination of sulfite levels and its oxidation in plant leaves. Plant Sci. 190, 123–130 (2012). 22608526 10.1016/j.plantsci.2012.04.004

[b73] Saeed A. I. . TM4a free, open-source system for microarray data management and analysis. Biotechniques 34, 374–378 (2003). 12613259 10.2144/03342mt01

[b74] Meyer A., Eskandari S., Grallath S. & Rentsch D. AtGAT1, a high affinity transporter for gamma-aminobutyric acid in *Arabidopsis thaliana*. J. Biol. Chem. 281, 7197–204 (2006). 16407306 10.1074/jbc.M510766200 PMC3009663

[b75] Baum G., Chen Y., Arazi T., Takatsuji H. & Fromm H. A plant glutamate decarboxylase containing a calmodulin binding domain. Cloning, sequence, and functional analysis. J. Biol. Chem. 268, 19610–19617 (1993). 8366104

[b76] Michaeli S. . A mitochondrial GABA permease connects the GABA shunt and the TCA cycle, and is essential for normal carbon metabolism. Plant J. 67, 485–498 (2011). 21501262 10.1111/j.1365-313X.2011.04612.x

