# Supplementary material for: Paclobutrazol induces tolerance in tomato to deficit irrigation through diversified effects on plant morphology, physiology and metabolism
Source: Sci Rep. 2016 Dec 22;6:39321. doi: 10.1038/srep39321 (PMC5177942; doi:10.1038/srep39321)
Supplement: Supplementary Information [file srep39321-s1.doc]

**Title: Paclobutrazol induces tolerance in tomato to deficit irrigation through diversified effect on plant morphology, physiology and metabolism**

Sikander Pal^#1α*^, Jiangsan Zhao^#1ω^, Asif Khan^#1§^, Narendra Singh Yadav^1^, Albert Batushansky^1β^, Simon Barak^1^, Boris Rewald^1ω^, Aaron Fait^1^, Naftali Lazarovitch^1^ and Shimon Rachmilevitch^1*^

**Supporting Method**

*Metabolomics*

An extensive metabolomic analysis was performed on the first and second harvest of tomato plants (leaves) with and without Pbz application and with/and without drought stress using a Thermo Scientific DSQ II GC/MS using a Factor Four Capillary VF-5ms column (Lise*c et al*., 2006).

**Metabolome profiling**

Tomato leaves of 105 days old plants subjected to irrigated and non-irrigated conditions (500 mg fresh weight, snap frozen in liquid N_2_ and stored at -80 °C till further analysis) were used extracted for metabolite analysis using a Thermo Scientific DSQ II GC/MS using a FactorFour Capillary VF-5ms column (Lise*c et al*., 2006). For each condition, six biological replicates from six independent plants were used. Obtained chromatograms were analyzed using Xcalibur software (version 2.0.7), and the level of metabolites was calculated by normalizing the intensity of the peak of each metabolite to the ribitol standard. ANOVA and student t-tests were performed using MEV 4.8.1 software (Saeed *et al.,* 2003).

**0.14**

**0.12**

**0.100**

**0.08**

**A**

**B**

**A**

***b***

**0.06**

***a***

***a***

**0.04**

**0.02**

**0.00**

**0-I**

**0.8-I**

**1.6-I**

**1.6-DI**

**0.8-DI**

**0-DI**

**Figure S1 Effects of Pbz application (0, 0.8 and 1.6 ppm) in irrigated (I) and deficit-irrigated (DI) tomato plants on the relative growth rate (RGR).** *Capital letters (*A*,*B*) indicate significant differences from the Pbz untreated control in the irrigated plants. Small italicized letters (a, b) indicate significant differences from the PBZ untreated control in the deficit-irrigated plants (one way ANOVA, p<0.05).*

**0-I**

**Onset of drought**

**Last measurement**

**0.8-I**

**0-DI**

**1.6-I**

**Chl. content index**

**0.8-DI**

**1.6-DI**

**Figure S2 Effects of Pbz application (0, 0.8 and 1.6 ppm) on the chlorophyll content index in irrigated (I) and deficit-irrigated (DI) tomato plants grown over a period of 105 days*.*** *Chl measurement started from 43 days (d) and extended upto 98 days after planting.*

**Specific leaf area (cm^2^g^-1^)**

**0-DI**

**0.8-DI**

**1.6-DI**

**0-I**

**0.8-I**

**1.6-I**

**Figure S3 Effects of Pbz application (0, 0.8 and 1.6 ppm) on the specific leaf area (SLA) in irrigated (I) and deficit-irrigated (DI) tomato plants**. *Capital letters (*A,B*) indicate significant differences from the Pbz untreated control in the irrigated plants. Small italicized letters (a, b) indicate significant differences from the Pbz untreated control in the deficit-irrigated plants (one way ANOVA, p<0.05).*

**
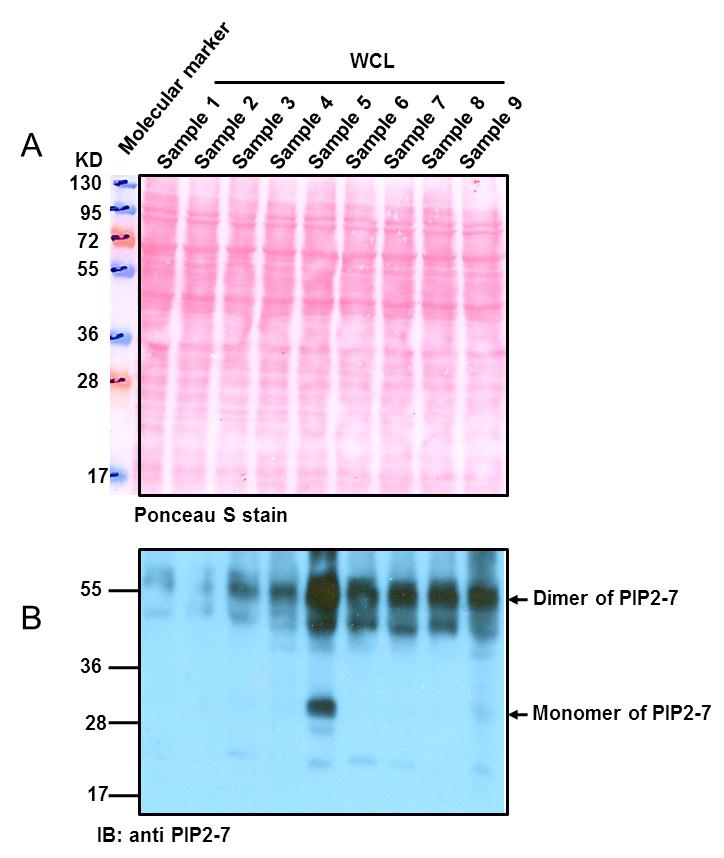
**

**C**

**A**

**
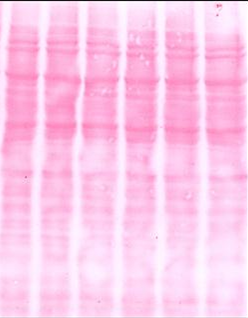
**

**M**

**0-I**

**0.8-I**

**1.6-I**

**0.8-DI**

**1.6-DI**

**0-DI**

**Relative gene expression**

**3. 5**

**1.0**

**1. 5**

**2. 5**

**3.0**

**2. 0**

**0-I 0.8-I 1.6-I 0-DI 0.8-DI 1.6-DI**

**0.0**

**0.** **5**

**
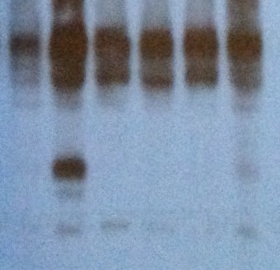
**

**B**

**Fig. S4 (A-C)** Effects **of Pbz application (0, 0.8 and 1.6 ppm) in irrigated (I) and deficit-irrigated (DI) tomato leaves on the expression of PIP2-7 aquaporin protein.** *A: showing molecular markers (M) for uniform protein loading, B: expression of PIP2-7 protein (normal water regime (data not shown), samples 1-3, irrigated plants, samples 4-6 deficit-irrigated plants ,C: relative gene expression of SlTIP2.*

**35**

**Leaf electrolyte %**

**25**

**30**

**20**

**15**

**10**

**0**

**5**

**67. 5**

**60**

**Root electrolyte %**

**45**

**52.5**

**37. 5**

**30**

**22. 5**

**15**

**7. 5**

**0-I 0.8-I 1.6-I 0-NI 0.8-NI 1.6-NI**

**0.02**

**0-I 0.8-I 1.6-I 0-DI 0.8-DI 1.6-DI**

**Figure S5 Effects of Pbz application (0, 0.8 and 1.6 ppm) in irrigated (I) and deficit-irrigated (DI, drought) on leaf electrolyte leakage (a) and root electrolyte leakage (b) in tomato plants**.  *Capital letters (*A,B*) indicate significant differences from the control* in irrigated (I) tomato plants*. Small italicized letters (a, b) indicate significant differences from the control in* deficit-irrigated (DI) tomato plants *(one way ANOVA, p<0.05).*

**Table S1: List of gene specific primers used for Real time-quantitative PCR analysis.**

| **Gene ID** (accession no.) | | **Forward primer (5’ 🡪3’)** | **Reverse primer (5’ 🡪 3’)** | | | | **Reference** |  | |
| --- | --- | --- | --- | --- | --- | --- | --- | --- | --- |
| **TCA cycle** | | | | | | | |  | |
| ***MtCS*** | TTGGATTGCCGCTAGAGAGG | | TTGCACTGCTTCTCAAGCCA | | Sienkiewicz-Porzucek, 2009 | | |  | |
| ***Aco1***(*Solyc07g052340*) | GCCGCTTGCTTCAACTTCTAC | | GACTCCACCTCGGCACAGA | | Morgan *et al.,* 2013 | | |  | |
| ***Aco2*** (*Solyc12g005860*) | TGGTGCTTATTGCTCTAGTGGGTA | | CAACACCGTATCTCCACCTCA | | Morgan *et al.,* 2013 | | |  | |
| ***SCoAL1***(AY167586.1) | GACCAAACTGATCGCAAATCTGTC | | CTTGGGTGTCACTCCACCAACC | | designed | | |  | |
| ***SCoAL2*** (AY650029.1) | GGCATCAGTATCGCTACTTTGGATC | | CTTGGGTGTCACTCCACCAACC | | designed | | |  | |
| ***SDH2*** | GCAGGTCCAACCTCAAGTC | | GGTTATCTGGATTCCATCGG | | Arau´ jo *et al.,* 2011 | | | |  |
| ***Fum1*** (BT013385.1) | AAGGTGAACATGGAGTATGGCCT | | CATCACCGCTTTCCCTATAGATG | | designed | | | |  |
| ***Fum 2*** (AK325138.1) | CTGAAAATGAACCTGGAAGCAGT | | GAGTAGGATTTACCTTGCCAGGC | | designed | | | |  |
| **GSH-ASC cycle** | | | | | | | | |  |
| ***DHAR1*** | AGGTGGCTCTTGGACACTTC | | CTTCAGCCTTGGTTTTCTGG | | | Mellidou *et al.,* 2012 | |  | |
| ***MDHAR1*** | CAAGGGTTTCGGTTCCTTCT | | CTGCATTTCCTCCTCCAACT | | | Mellidou *et al.,* 2012 | |  | |
| ***GR*** | TTGGTGGAACGTGTGTTCTT | | TCTCATTCACTTCCCATCCA | | | Ahammed *et al.,* 2013 | | | |
| ***APX*** | TCTGAATTGGGATTTGCTGA | | CGTCTAACGTAGCTGCCAAA | | | Ahammed *et al.,* 2013 | | | |
| ***PG-beta*** | GATCGGCGATGCATTGAGTGAGT | | CATTTGATCCTTTTGTATCCTCAGTCG | | | Sun *et al.,* 2012 | | | |
| ***GLDH*** | TCGAGTTCAGCAGCTTGTGGATGA | | CACCAACCTGAACAATGCCACCAA | | | Alimohammadi *et al.,* 2007 | | | |
| **Sugar metabolism** | | | | | | | | | |
| ***HK2*** | | CAGCTGGTATCTTGGGCATT | ACCATCATTCGCGTGCTT | | Kortstee *et al.,* 2007 | | | | |
| ***HK1*** | | CCAATGCAGCTTATGTGGAAC | CATGCCAGAAGTCATCTTCTCA | | Kortstee *et al.,* 2007 | | | | |
| ***FK2*** | | TTGTTGGTGCCCTTCTAACCA | ACGATGTTTCTATGCTCCTCCCT | | Kortstee *et al.,* 2007 | | | | |
| ***FK1*** | | CTCCGTTACATATCTGATCCTT | GACAGCATTGAAGTCACCTT | | Kortstee *et al.,* 2007 | | | | |
| ***SUS3*** | | GAACGAAGAACACCTGTGTGT | TCTCTGCCTGCTCTTCCAA | | Kortstee *et al.,* 2007 | | | | |
| ***SUS2*** | | TTTGGCTGACTGTTGTCGAAG | GTCGAAATGGTTTCCCAATG | | Kortstee *et al.,* 2007 | | | | |
| ***Pgm*** | | TAGTTGCACGTATGGGATTGTC | GGCAACACCTTTCAAACCT | | Kortstee *et al.,* 2007 | | | | |
| **GABA metabolism** | | | | | | | | | |
| ***GAD2*** | | CTTTGATCTTCTCCGTCGTTG | ATATCGAGACGCGAAAGTCG | | Mae *et al.,* 2012 | | | | |
| ***GAD1*** | | AAACTTCCCATTTCCCAACC | CGATTGATCGGAGGAGAAAA | | Mae *et al.,* 2012 | | | | |
| ***GABAT1*** | | CAGCACAAGCTTGACGATGG | TGGTGATTACTGGTTAAGGG | | Koike *et al.,* 2013 | | | | |
| **Cell wall enzymes** | | | | | | | | | |
| ***PME*** | | TGGTTACAGCACAAGGTCGT | CTGACCCACAGTGAATTTCG | | Chapman *et al.,* 2012 | | | | |
| ***XTH5*** | | GGATTCAGCCATCTCTTTGGTG | GAACCCTGAACCTGTGTTTTGG | | Centeno *et al.,* 2011 | | | | |
| ***EXP1*** | | TACCAATTTCTGCCCACCAAAT | GGTTACACCAGCCACCATTGT | | Centeno *et al.,* 2011 | | | | |
| **ABA metabolism** | | | | | | | | | |
| ***AAO1*** | | CCAGGCACAAACACAATCAA | GTCGTAAATAATATCAGACTG | | Horváth *et al.,* 2011 | | | | |
| ***ZEP*** | | GGTCGTGTTACATTGCTTGG | TGCATGCTTTTTCAAGTTCC | | Kilambi *et al.,* 2013 | | | | |
| ***NCED1*** | | CTTATTTGGCTATCGCTGAACC | CCTCCAACTTCAAACTCATTGC | | Nitsch *et al.,* 2009 | | | | |
| **Polyamine** | | | | | | | | | |
| ***SPS*** (AJ006414) | | TGCTTGTTTTGGATGGTGTG | GCAGGTCCCTCAGTAGAGCA | | | Tassoni *et al.,* 2006 | | | |
| **Aquaporins** | | | | | | |  | | |
| ***TIP2*** (EST512332) | ACTGGATTGCTCAACTTTTGGGT | | | CTTTGAGAAGGTAACAGCCAACAA | | | designed | | |
| **Endogenous reference gene** | | | | | | |  | | |
| ***Actin Tom 41***(U60480) | | CATGCCATTCTCCGTCTTGA | CGCTCGGTCAGGATCTTCAT | | | | Yarmolinsky *et al.,* 2013 | | |

*MtCS*, mitochondrial citrate synthase; *Aco1*, aconitase1; *ScoAL1*, succinyl CoA-ligase; *SDH*, succinic dehydrogenase; *Fum1*, fumarase1; *DHAR1*, dehydro ascorbate reductase; *MDHAR*, mono DHAR; GR, glutathione reductase; *APX*, ascorbate peroxidase; *PG-beta*, polygalaturonase-beta; *GLDH*, L-galactono-g-lactonedehydrogenase; *HK*, hexokinase; *FK*, fructokinase; *SUS*, sucrose synthase; *GAD*, glutamate decarboxylase; *GABA-T*, GABA-transaminase; PME, pectin methylesterase; *XTH5*, xyloglucan endotransglycosylase 5; *EXP1*, expansin1; AAO1, ABA aldehyde oxidase; *ZEP*, zeaxanthin epoxidase; *NCED1*, 9-*cis*-epoxy-carotenoid dioxygenase; *SPS*, spermine synthase, *TIP2*,  tonoplast intrinsic protein2 gene family.

**References**

1. Lisec, J. *et al.* Gas chromatography mass spectrometry–based metabolite profiling in plants. *Nat. Protoc.* **1,** 387-396 (2006).
2. Saeed, A. I. *et al.* TM4a free, open-source system for microarray data management and analysis. *Biotechniques* **34,** 374-378 (2003).
3. Sienkiewicz-Porzucek, A. *Evaluation of the role of mitochondrial citrate synthase, mitochondrial and cytosolic isoforms of isocitrate dehydrogenase in tomato leaf metabolism*. PhD thesis, Humboldt-University, Berlin (2009).
4. Morgan, M. J. *et al*. Metabolic engineering of tomato fruit organic acid content guided by biochemical analysis of an introgression line. *Plant Physiol.* **161,** 397-407 (2013).
5. Araújo, W. L. *et al.* Antisense inhibition of the iron-sulphur subunit of succinate dehydrogenase enhances photosynthesis and growth in tomato via an organic acid–mediated effect on stomatal aperture. *Plant Cell* **23,** 600-627 (2011).
6. Mellidou, I., Keulemans, J., Kanellis, A. K. & Davey, M. W. Regulation of fruit ascorbic acid concentrations during ripening in high and low vitamin C tomato cultivars. *BMC Plant Biol.* **12,** 239 (2012).
7. Ahammed, G. J. *et al*. Role of brassinosteroids in alleviation of phenanthrene–cadmium co-contamination-induced photosynthetic inhibition and oxidative stress in tomato. *J. Exp. Bot.* **64,** 199-213 (2013).
8. Sun, L. *et al*. Suppression of 9-cis-epoxycarotenoid dioxygenase, which encodes a key enzyme in abscisic acid biosynthesis, alters fruit texture in transgenic tomato. *Plant Physiol.* **158,** 283-298 (2012).
9. Alimohammadi, M., de Silva, K., Ballu, C., Ali, N. & Khodakovskaya, M. V. Reduction of inositol (1, 4, 5)–trisphosphate affects the overall phosphoinositol pathway and leads to modifications in light signalling and secondary metabolism in tomato plants. *J. Exp. Bot.* **63,** 825-835 (2012).
10. Kortstee, A. J., Appeldoorn, N. J. G., Oortwijn, M. E. P. & Visser, R. G. F. Differences in regulation of carbohydrate metabolism during early fruit development between domesticated tomato and two wild relatives. *Planta* **226,** 929-939 (2007).
11. Mae, N. *et al*. Accumulation mechanism of γ-aminobutyric acid in tomatoes (*Solanum lycopersicum* L.) under low O2 with and without CO2. *J. Agri. Food Chem.* **60,** 1013-1019 (2012).
12. Koike, S., Matsukura, C., Takayama, M., Asamizu, E. & Ezura, H. Suppression of γ-aminobutyric Acid (GABA) transaminases induces prominent GABA accumulation, dwarfism and infertility in the tomato (*Solanum lycopersicum* L.). *Plant Cell Physiol.* **54,** 793-807 (2013).
13. Chapman, N. H. *et al*. High-resolution mapping of a fruit firmness-related quantitative trait locus in tomato reveals epistatic interactions associated with a complex combinatorial locus. *Plant Physiol.* **159,** 1644-1657 (2012).
14. Horváth, E., Gallé, Á., Szepesi, Á., Tari, I., & Csiszár, J. Changes in aldehyde oxidase activity and gene expression in *Solanum lycopersicum* L. shoots under salicylic acid pre-treatment and subsequent salt stress. *Acta Biologica Szegediensis* **55,** 83-85 (2011).
15. Kilambi, H. V., Kumar, R., Sharma, R. & Sreelakshmi, Y. Chromoplast-specific carotenoid-associated protein appears to be important for enhanced accumulation of carotenoids in hp1 tomato fruits. *Plant Physiol.* **161,** 2085-2101 (2013).
16. Nitsch, L. M. C. *et al*. Abscisic acid levels in tomato ovaries are regulated by *LeNCED1* and *SlCYP707A1*. *Planta* **229,** 1335-1346 (2009).
17. Tassoni, A., Watkins, C. B., & Davies, P. J. Inhibition of the ethylene response by 1-MCP in tomato suggests that polyamines are not involved in delaying ripening, but may moderate the rate of ripening or over-ripening. *J. Exp. Bot.* **57,** 3313-3325 (2006).
18. Yarmolinsky, D., Brychkova, G., Fluhr, R., & Sagi, M. Sulfite reductase protects plants against sulfite toxicity. *Plant Physiol.* **161,** 725-743 (2013).
